# Small bowel neuroendocrine tumor presenting with chronic diarrhea and mesenteric ischemia: A case report

**DOI:** 10.1002/ccr3.9508

**Published:** 2024-10-31

**Authors:** Bisher Sawaf, Shahem Abbarh, Ashraf I. Ahmed, Malik Halabiya, Abdellatif Ismail, Souraia Mezhoud

**Affiliations:** ^1^ Department of Internal Medicine, Division of Gastroenterology and Hepatology Hamad Medical Corporation Doha Qatar; ^2^ Department of Internal Medicine University of Toledo Medical Center Toledo Ohio USA; ^3^ Department of Internal Medicine MedStar Health Baltimore Maryland USA; ^4^ College of Medicine, QU Health Qatar University Doha Qatar; ^5^ Department of Internal Medicine University of Maryland Medical Center, Midtown Campus Baltimore Maryland USA

**Keywords:** carcinoid, chronic diarrhea, gastrointestinal neuroendocrine tumors, mesenteric ischemia, NETs, small intestine

## Abstract

**Key Clinical Message:**

The diagnosis of gastrointestinal neuroendocrine tumors is often challenging owing to the nonspecific presentation. This may lead to delayed diagnosis and serious, rare complications, such as acute mesenteric ischemia. This case highlights the importance of early recognition and the need for a multidisciplinary approach to managing such cases.

**Abstract:**

Gastrointestinal (GI) neuroendocrine tumors (NETs) are rare neoplasms originating from neuroendocrine cells within the digestive tract. Despite their rarity, their incidence is increasing, necessitating a better understanding of their presentation and management. In the present report, we present a case of duodenal bulb NET that caused chronic diarrhea and unintentional weight loss for 2 years before manifesting as acute mesenteric ischemia. This case sheds light on the diagnostic challenges associated with GI NETs, particularly in cases with nonspecific symptoms. In addition, it underscores the importance of prompt recognition and management, as evidenced by the progression of the patient to acute mesenteric ischemia.

## Graphical abstract

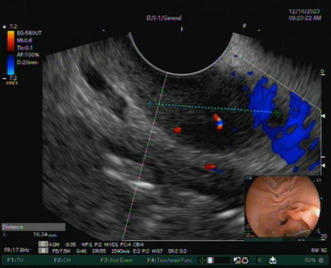

**Abbreviations**
CBC: complete blood count
CRP: C‐reactive protein
CT: computed tomography
EUS: endoscopic ultrasound
GI: gastrointestinal
MDT: multidisciplinary team
MRI: magnetic resonance imaging
NETs: neuroendocrine tumors
PET: positron emission tomography
PRRT: peptide receptor radionuclide therapy
SRS: somatostatin receptor scintigraphy
WHO: World Health Organization

## 1 INTRODUCTION

Gastrointestinal (GI) neuroendocrine tumors (NETs) are rare neoplasms originating from neuroendocrine cells within the digestive tract.^1^ Several epidemiological studies showed an overall increased incidence of NETs worldwide, particularly in North America, over the past few decades.^2^, ^3^ The World Health Organization (WHO) classifies NETs into well‐differentiated and poorly differentiated. Well‐differentiated NETs are further divided into low‐grade (G1), intermediate‐grade (G2), and high‐grade (G3) subtypes based on proliferative rate, while poorly differentiated NETs are considered G3 carcinomas.^4^

NETs present with a spectrum of site‐specific symptoms such as gastric ulcers, abdominal pain, nausea, vomiting, and constipation. Additionally, patients may exhibit carcinoid syndrome symptoms characterized by flushing, diarrhea, and bronchospasm. In severe cases, carcinoid crisis, typically triggered by exposure to anesthetic agents or manipulation of the tumor during biopsy or surgery, can lead to sudden hemodynamic instability.^5^ Although GI NETs can affect individuals of all ages, they commonly affect individuals older than 50 years old.^6^

NETs can manifest in various GI sites, including the small intestine, rectum, appendix, colon, and stomach.^5^ The diagnosis of GI NETs involves a comprehensive approach incorporating imaging modalities such as computed tomography (CT) scan, magnetic resonance imaging (MRI), and somatostatin receptor scintigraphy (SRS).^7^ Treatment strategies are tailored based on factors such as tumor stage, tumor grade, and metastasis. Overall, surgical resection is the main approach in managing large type III GI NETs.^8^ On the contrary, the endoscopic therapy approach is increasingly applied, particularly with new techniques, including endoscopic mucosal resection and endoscopic submucosal dissection.^9^ In addition, advanced presentations may occasionally necessitate systemic interventions such as somatostatin analogs, targeted therapies, or peptide receptor radionuclide therapy (PRRT).^7^ This paper reports a rare case of a small‐bowel neuroendocrine tumor presenting with acute intestinal ischemia.

## 2 CASE PRESENTATION

### 2.1 Presentation and investigation

A 58‐year‐old Asian woman presented with a 2‐year history of chronic watery diarrhea. She also reported occasional diffuse crampy abdominal pain and unintentional weight loss of 8 kg over the past year. Notably, the patient denied any symptoms of fever, blood in the stool, joint pain, skin rash, visual symptoms, or Raynaud's symptoms. The patient had multiple previous visits to the Emergency Department due to episodes of profuse, non‐bloody diarrhea and dehydration that were managed symptomatically. Otherwise, the patient had no significant medical or surgical history.

**FIGURE 1:**
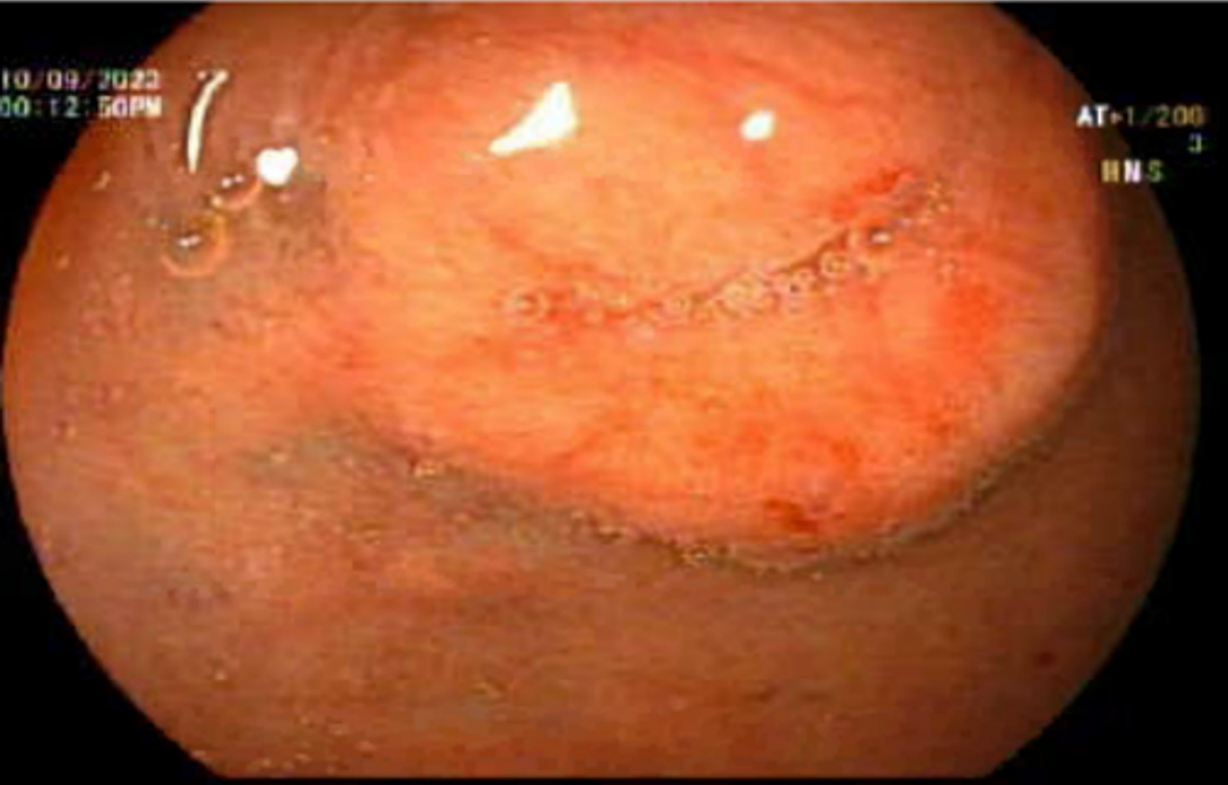
Endoscopic view of a small subepithelial polypoid lesion located at the anterior wall of the duodenal bulb.

**FIGURE 2:**
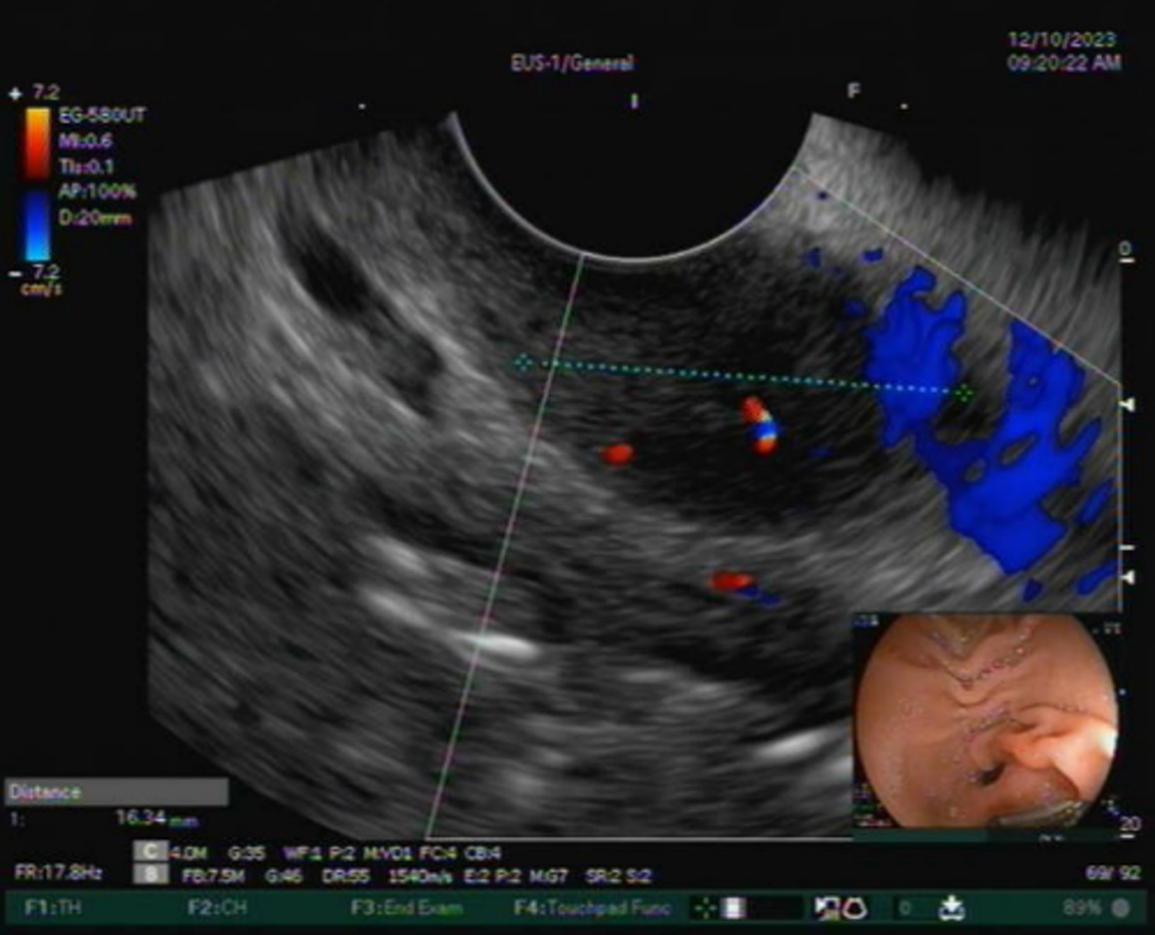
Endoscopic ultrasound showing a well‐defined subepithelial lesion of 16 mm diameter with internal vascularity located at the duodenal bulb.

Systemic examinations were unremarkable, except for a body mass index of 18. The patient was initially investigated in an outpatient setting. Laboratory tests to rule out infectious causes, inflammatory bowel disease, hyperthyroidism, and celiac disease revealed a normal complete blood count (CBC), complete metabolic panel, C‐reactive protein (CRP), thyroid function tests, and celiac serology. The stool cell count, electrolytes, culture, ova and parasite, and calprotectin were also unrevealing. Upper endoscopy revealed a small subepithelial polypoid lesion with a central depression, not ulcerated, located at the anterior wall of the duodenal bulb (Figure 1). A colonoscopy revealed congestion of the ileocecal valve, with a few superficial ulcers and an edematous terminal ileum containing a few aphthous ulcers. Biopsies from the stomach, duodenum, terminal ileum, and colon were unremarkable, with no evidence of active inflammation or granuloma. A CT enteroclysis showed enhanced mucosal thickening in the terminal ileum without mesenteric or retroperitoneal lymphadenopathy. Endoscopic ultrasound (EUS) showed a well‐defined subepithelial lesion of 16 mm diameter with internal vascularity (Figure 2). Fine‐needle aspiration of the lesion revealed a well‐differentiated neuroendocrine tumor, grade I, Stage II, with the following immunohistochemistry results: positive synaptophysin, chromogranin, and cytokeratin AE1/AE3, with a KI 67 index of less than 2%. Serum chromogranin and gastric levels were high, whereas the serotonin level was within the normal range. The patient was planned for a gallium Ga‐68 DOTATATE‐integrated positron emission tomography (PET)/CT scan.

**FIGURE 3:**
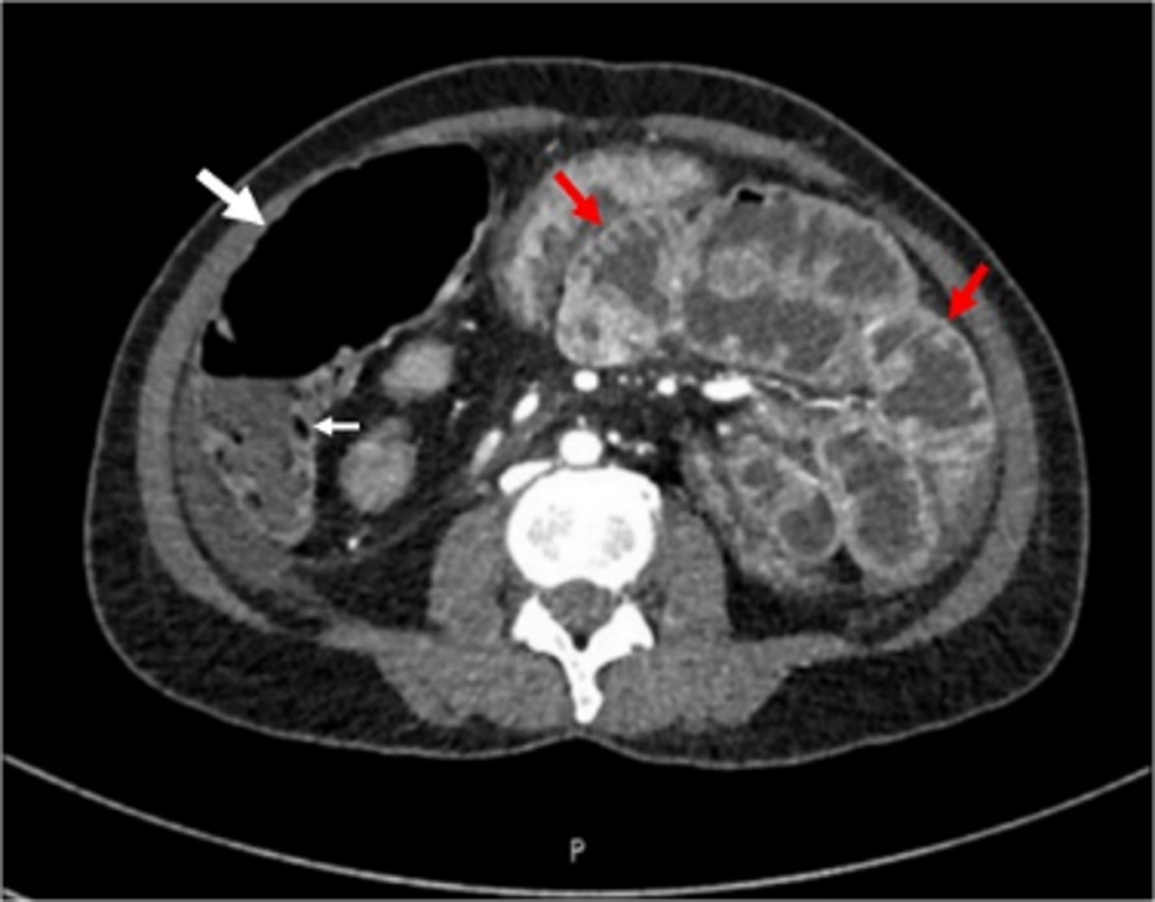
Computed tomography scan of the abdomen with contrast revealing mesenteric ischemia, with pneumatosis intestinalis (white arrows) and small bowel wall enhancement (red arrows).

However, before the scheduled DOTATATE scan, the patient was admitted with acute onset of severe abdominal pain that happened 2 weeks after NET diagnosis. The patient's initial vital signs were normal. She rapidly developed severe hypotension and tachycardia. Investigations revealed a five‐gram drop in hemoglobin, a high lactic acid level, and a lipase level of 503 U/L (the normal value is 0–160 U/L). An urgent contrast‐enhanced CT scan of the abdomen revealed evidence of mesenteric ischemia in the form of pneumatosis intestinalis and portal venous gas, with small bowel wall enhancement (Figure 3). The pancreas showed normal enhancement with no areas of necrosis or cystic changes; however, air foci were observed in the pancreatic head.

### 2.2 Treatment

After resuscitation, the patient underwent urgent diagnostic laparoscopy, which was converted to exploratory laparotomy. A patchy gangrenous segment involving the distal jejunum until the mid‐ileum was resected. The proximal jejunum (150 cm from the duodenojejunal junction) and distal ileum (55 cm from the ileocecal valve) were preserved. Subsequent histopathological examination of the resected bowel segment, measuring 45 cm in length, confirmed acute transmural inflammation and necrosis consistent with ischemic bowel, extending to the surgical margins. Two days later, the patient underwent re‐loop laparotomy, with the creation of a double‐barrel stoma and abdominal closure.

### 2.3 Outcome and follow‐up

The patient's clinical condition improved slightly within the next few days; however, a week postoperatively, she experienced another drop in hemoglobin level and hypotension. Workup to rule out cardiac embolic source, including transthoracic echocardiogram and telemetry, was unremarkable. A repeat CT scan of the abdomen with contrast showed reduced caliber of the entire length of the superior mesenteric artery and its branches, suggesting vasculitis versus vasospasm secondary to hypotension. Upper endoscopy showed a non‐bleeding ulcer with an adherent clot (Forrest IIb) located on the junction between the first and second parts of the duodenum. She was managed with intravenous esomeprazole and supportive blood transfusions. An extensive autoimmune workup to investigate the possibility of mesenteric vasculitis was inconclusive and was deemed less likely after a multidisciplinary team (MDT) meeting. Subsequently, a Ga‐68 DOTATATE‐integrated PET/CT scan revealed somatostatin receptor‐positive disease in the duodenum and a tiny nodule in the perigastric region without evidence of metastasis to the liver or extrahepatic sites. Another MDT with upper GI surgery was performed and concluded for distal gastrectomy once the patient was more stable. However, the patient had recurrent infections and passed away 2 months after hospitalization.

## 3 DISCUSSION

NETs represent a heterogeneous group of neoplasms arising from neuroendocrine cells, primarily affecting the GI tract.^1^ Small intestine neuroendocrine tumors, originating from intestinal enterochromaffin cells, typically demonstrate serotonin immunoreactivity and are frequently diagnosed around the age of 50.^6^ Despite their slow growth pattern, the diagnosis of NETs is often delayed due to the absence of specific clinical manifestations, except in cases of carcinoid syndrome, and the lack of definitive blood biomarkers.^10^ In this case, the subtle presentation of prolonged diarrhea was the only clue prior to her presentation with abdominal pain.

Mesenteric metastases are prevalent in small‐intestine NETs, primarily affecting the regional mesenteric and para‐aortic lymph nodes, with a high incidence observed in surgical cohorts. These metastases often coexist with liver involvement, which may precipitate carcinoid syndrome characterized by flushing, diarrhea, bronchoconstriction, and cardiac manifestations due to the systemic release of vasoactive substances.^11^ However, the absence of liver disease, as observed in this case, can contribute to the insidious clinical course of the disease.

Clinical manifestations of small intestine carcinoid tumors vary widely, with common symptoms including postprandial and colicky abdominal pain, gastrointestinal bleeding, obstruction, and anorexia.^1^ However, a considerable proportion of patients may remain asymptomatic. Catastrophic presentations, such as carcinoid abdominal crises, can occur and are characterized by intestinal ischemia resulting from vascular compromise due to mesenteric lymph node infiltration or hormone‐induced elastic vascular sclerosis.^12^, ^13^ Establishing a diagnosis of NETs poses significant challenges, often necessitating extensive and costly diagnostic evaluations.^14^ Imaging modalities such as CT angiography, somatostatin receptor scintigraphy, and PET imaging with radiolabeled serotonin precursors play pivotal roles in diagnosis and staging.^15^

The discussion extends to the intricacies of mesenteric ischemia as a rare yet severe complication of small bowel NETs, often associated with mesenteric metastasis, and the mechanisms, including cytokine‐induced fibrosis and neuroimmune responses, underscore the complexity of ischemic events.^16^ Chronic mesenteric ischemia, usually secondary to atherosclerotic disease and typically presenting with postprandial pain and weight loss, often underlies acute mesenteric arterial thrombosis. This process frequently occurs at the origin of visceral arteries, notably affecting the superior mesenteric artery and often accompanied by celiac artery occlusion.^17^ Other common causes of acute mesenteric arterial occlusion include embolic obstruction, frequently secondary to atrial fibrillation, valvular disease, or recent myocardial infarction.^18^ Mesenteric venous thrombosis, comprising less than 10% of mesenteric infarction cases, arises from stagnant blood flow, hypercoagulability, and endothelial damage, often without discernible triggers, particularly in young patients, with contributing factors including inherited diseases, surgical trauma, and emerging evidence indicating fibrinolysis shutdown as a significant risk factor.^18^ Acute nonocclusive mesenteric ischemia, observed in approximately 20% of cases, is characterized by superior mesenteric artery vasoconstriction due to diminished splanchnic blood flow, affecting not only the proximal colon but also often coinciding with severe coexisting conditions, such as cardiac failure or sepsis, where hypovolemia or vasoconstrictive agents can serve as triggering factors.^18^, ^19^

Treatment strategies for GI NETs differ based on several factors, including tumor size, site, grade, and metastasis status. Surgical resection remains the primary approach for localized aggressive lesions, offering potential curative outcomes with a 5‐year survival rate ranging from 80% to 100%.^20^ The endoscopic treatment approach, including new techniques such as endoscopic mucosal resection and endoscopic submucosal dissection, is increasingly used. The endoscopic option has been demonstrated to be effective and less invasive when used in the appropriate clinical context.^9^ Additionally, medical therapies such as somatostatin analogs, interferon‐alpha, and peptide receptor radionuclide therapy have been employed for symptom control and tumor stabilization.^21^, ^22^ Emerging therapeutic avenues, including targeted agents such as everolimus and sunitinib, are under investigation, particularly for well‐differentiated pancreatic NETs.^23^ Regular monitoring with biochemical markers and imaging studies is essential for assessing treatment response and disease progression.^20^ The evolution of treatment guidelines and the advent of novel therapeutic modalities have substantially improved patient outcomes, with median survival exceeding 16 years in specialized centers of excellence.^24^

## 4 CONCLUSION

This case highlights the challenges in diagnosing and treating GI NETs, especially those with subtle presentations and rare complications, such as mesenteric ischemia. Early identification of atypical symptoms is crucial. Mesenteric ischemia, though rare, is life‐threatening in small‐bowel NETs, requiring prompt multidisciplinary intervention. Vigilance in recognizing rare complications, such as mesenteric ischemia, is vital for enhancing patient care and refining treatments.

## AUTHOR CONTRIBUTIONS

**Bisher Sawaf:** Conceptualization; data curation; formal analysis; methodology; validation; visualization; writing – original draft; writing – review and editing. **Shahem Abbarh:** Conceptualization; formal analysis; investigation; methodology; validation; visualization; writing – original draft; writing – review and editing. **Ashraf I. Ahmed:** Conceptualization; formal analysis; validation; visualization; writing – original draft; writing – review and editing. **Malik Halabiya:** Conceptualization; validation; visualization; writing – original draft; writing – review and editing. **Abdellatif Ismail:** Conceptualization; validation; visualization; writing – original draft; writing – review and editing. **Souraia Mezhoud:** Conceptualization; data curation; supervision; validation; visualization; writing – original draft; writing – review and editing.

## FUNDING INFORMATION

This research did not receive a specific grant from funding agencies in the public, commercial, or not‐for‐profit sectors.

## CONFLICT OF INTEREST STATEMENT

The authors report no conflict of interest.

## ETHICS STATEMENT

The article was approved by the Institution Review Board at Hamad Medical Corporation.

## CONSENT

Written informed consent was obtained from the patient to publish this report in accordance with the journal's patient consent policy.

## Data Availability

The data that support the findings of this study are available from the corresponding author upon reasonable request.
